# Epigenetic Combinatorial Patterns Predict Disease Variants

**DOI:** 10.3389/fgene.2017.00071

**Published:** 2017-05-30

**Authors:** Yu Zhang

**Affiliations:** Department of Statistics, Pennsylvania State UniversityUniversity Park, PA, United States

**Keywords:** epigenetics, cell-type specificity, functional mutation, GWAS, Bayesian method

## Abstract

Most genetic variants identified in genome-wide association studies are noncoding and are likely tagging nearby causal variants. It is a challenging task to pinpoint the precise locations of disease-causal variants and understand their functions in disease. A promising approach to improve fine mapping is to integrate the functional data currently available on hundreds of human tissues and cell types. Although there are several methods that use functional data to prioritize disease variants, they mainly use linear models, or equivalent naive likelihood-based models for prediction. Here, we investigate whether study of the combinatorial patterns of functional data across cell types can improve prediction accuracy for disease variants. Using functional annotation in 127 human cell types, we first introduce a Bayesian method to identify recurring cell-type-specificity partitions on the scale of the genome. We show that our *de novo* identification of epigenome partition patterns agrees well with known cell-type origins and that the associated functional elements are strongly enriched in disease variants. Using epigenetic cell-type specificity in addition to enrichment of functional elements, we further demonstrate that the power to predict disease variants can be greatly improved over that achievable with linear models. Our approach thus provides a new way to prioritize disease functional variants for testing.

## Introduction

Genome-wide association studies (GWAS) have identified thousands of genetic variants associated with hundreds of complex human diseases. Most disease variants are located in noncoding regions (Welter et al., 2014), the functions of which are difficult to interpret. Several studies (Meyer et al., 2008; Maurano et al., 2012) have shown that disease variants are enriched in gene regulatory regions and that they may affect phenotypes at the regulatory level (Verlaan et al., 2009; Petronis, 2010; Stitzel et al., 2010; Schödel et al., 2012; French et al., 2013; Sharma et al., 2014). In addition, disease variants are likely to affect gene regulation and phenotypes in a cell-type-specific manner (Fu et al., 2012; Hardison, 2012; Rhie et al., 2013). Using massively parallel sequencing technologies, a plethora of data sets have been generated on a wide collection of functional marks in the human genome in many cell lines and primary *ex vivo* tissues. A major challenge is how to integrate these functional data in multiple cell types to pinpoint disease-causal variants and understand their molecular and organismal effects in a cell-type-specific context (Edwards et al., 2013; Kircher et al., 2014).

While many methods have used functional annotations to prioritize disease-causal variants (Pickrell, 2014; Farh et al., 2015; Kichaev and Pasaniuc, 2015; Li and Kellis, 2016), they have not considered the combinatorial effects of functional elements in different cell types for prediction. The most commonly used approach is based on linear models, where functional data on different epigenomic marks in one or more cell types are used as predictors in a regression model, and the GWAS *p*-values or the known disease risk variants are used as responses. There is an alternative approach using likelihood-based methods, but these methods simply add the likelihoods calculated from each cell type or each category of annotation to compute a total score for each genetic variant, and then use the total score to prioritize disease variants. This is equivalent to an additive model but ignores correlations between different annotations.

There are two major challenges in detecting and utilizing combinatorial epigenomic patterns across cell types for GWAS prediction. First, functional elements inferred in each cell type separately are subject to annotation errors (Roadmap Epigenomics Consortium). When compared across cell types, they will create a large number of spurious combinations of epigenomic patterns with low frequencies, which can substantially reduce the predictive power. Second, the number of possible combinations of functional annotations across cell types grows rapidly with the number of distinct functional elements and the number of cell types involved. Naively including all combinations in one model will over-fit the data and will not produce reliable results, owing to strong correlation between the combinations. For instance, with hundreds of cell types studied in the ENCODE (Encode Project Consortium, 2012) and Roadmap Epigenomics (Roadmap Epigenomics Consortium et al., 2015) projects, and tens of epigenetic states inferred in each cell type (Ernst and Kellis, 2012), the number of potential predictors to be included in a model can easily become intractable, even when just considering pairwise interactions. Existing studies have therefore only used either a subset of cell types most relevant to disease or a linear model across cell types for predicting disease variants (Li and Kellis, 2016).

In this work, we investigate whether combinatorial patterns of epigenomic data across different cell types can improve the power to predict disease variants. Using the 111 cell types from the Roadmap Epigenomics project and 16 ENCODE tier 1 and 2 cell types, we first apply IDEAS (Zhang et al., 2016) to re-annotate functional elements in the 127 epigenomes. IDEAS is a two-dimensional genome segmentation method that identifies *de novo* functional elements from multivariate epigenetic marks along the genome and across multiple cell types simultaneously. The IDEAS method is distinct from existing genome segmentation methods in that it borrows information both along the genome and across cell types, which leads to a gain in power because different cell types share the same underlying DNA sequences. As a result, IDEAS can produce more accurate and consistent functional annotations than other methods. Using the functional annotations as input, we next develop a Bayesian algorithm for *de novo* identification of distinct and recurring patterns of epigenome partition patterns in the whole genome. Each pattern of epigenome partitions represents a distinct nonlinear relationship between functional elements across cell types, where the functional elements in the cell types within the same partition have the same distribution, and thus captures cell-type specificity. Hereinafter, we refer to a specific configuration of epigenome partition as a CSP (cell-type-specificity pattern). Finally, we calculate enrichment scores of functional elements within each CSP and use both the CSP and epigenetic state enrichment scores as predictors for prioritizing disease variants. Notably, we do not make assumptions on the relationships between each cell type and the disease, since such information is often unknown. We evaluate the proposed method on 532 complex traits in the GWAS Catalog (Welter et al., 2014). We show that in a large number of complex traits, the disease variants are enriched in active functional elements, with this enrichment frequently being cell-type-specific and interpretable with respect to each trait. By comparing our results with those of linear models, we further show that incorporating nonlinear epigenetic CSPs can indeed improve the accuracy for predicting disease variants compared with the use of either a single best-matched cell type or all cell types in an additive way.

## Materials and methods

### Joint genome segmentation of the 127 epigenomes

We downloaded the *p*-value tracks of five histone marks (H3k4me3, H3k4me1, H3k36me3, H3k27me3, and H3k9me3) in 111 epigenomes from the NIH Roadmap Epigenomics project and 16 epigenomes from the ENCODE project. These five histone marks were the only marks commonly generated in all of the 127 epigenomes and were used by the Roadmap Epigenomics consortium to produce the first functional map in the 127 epigenomes. The *p*-value tracks of histone marks were calculated against the input data within the same cell types, thereby removing cell-type-specific bias and enabling comparison across cell types. We used the average log *p*-values per 200 bp window for each histone mark as input; this is the same window size as was used by the Roadmap Epigenomics Consortium and is appropriate for the wide spread of signals in histone marks. The data matrix contained 13,844,320 rows and 635 columns, consisting of 8.8 billion observations in total. All data were mapped to hg19.

We ran IDEAS (Zhang et al., 2016) to segment the 127 epigenomes jointly, which assigned epigenetic states to 200 bp sliding windows in each cell type, capturing the distinct combinatorial patterns of the five histone marks. We first ran IDEAS on chromosome 1, which produced a 25-state model. We then combined chromosome 1 with each of the other chromosomes and re-ran IDEAS, keeping the segmentation results of chromosome 1 unchanged. As a result, all other chromosomes were segmented conditionally independently, given chromosome 1. Our full model-based inference of functional elements in all epigenomes produced homogeneous and position-wise comparable state assignments across the 127 epigenomes, which was ideal for the proposed task of detecting position-wise CSPs. The whole-genome tracks of segmentations for the 25-state model can be accessed in the UCSC genome browser via a hub link to http://bx.psu.edu/~yuzhang/hub.txt.

### Detecting epigenetic cell-type specificity

We have developed a Bayesian method to identify recurring CSPs from the IDEAS segmentation. Let *X*_·*j*_ = {*X*_1, *j*_, …, *X*_*N,j*_} denote the states assigned to *N* epigenomes at positions *j* = 1, …, *L*, where *X*_*i,j*_ = 1, …, *S* denotes the state assigned to the *i*th epigenome at the *j*th position. We want to partition the *N* epigenomes into *K* groups at each position, where some groups may be empty, such that within each group the epigenomes have a common and position-dependent distribution of epigenetic states. We assume that the whole genome has *C* distinct CSPs, denoted by Ω = {Ω_1_, …, Ω_*C*_}. Each CSP specifies how the epigenomes are assigned to *K* groups. We further assume that each CSP occurs with probability *p*_*c*_ independently at each position, and we denote by *M*_*j*_ = 1, …, *C* the CSP at the *j*th position. We express the probability function as

(1) $$\begin{array}{l} P\left(\Omega ,M|X\right)\propto P\left(\Omega ,M,X\right)=\mathrm{Pr}\left(X|\Omega ,M\right)\mathrm{Pr}\left(\Omega ,M\right)\\ =\prod_{j}p_{M_{j}}\mathrm{Pr}\left(X_{\cdot j}|\Omega_{M_{j}}\right)\prod_{c=1}^{C}\text{Pr}\left(\Omega_{c}\right) \end{array}$$

where *p*_*Mj*_ denotes the prior probability of CSP *M*_*j*_, Pr(*X*_·*j*_|Ω_*M*_*j*__) denotes the state distribution function given Ω_*M*_*j*__ at position *j*, and Pr(Ω_*c*_) denotes the prior distribution of epigenome partitions in Ω_*c*_.

We assume a multinomial distribution for the states within each group of epigenomes in each CSP, with position-specific distribution parameters. Let *n*_*kjs*_ denote the number of states *s* observed in the *k*th group of epigenomes in Ω_*M*_*j*__ at position *j*. Using the Dirichlet($\overset{⇀}{a}$) prior for multinomial distributions, we obtain

(2) $$\begin{array}{l} \mathrm{Pr}\left(X_{\cdot j}|\Omega_{M_{j}}\right)=\prod_{k=1}^{K}\left(\frac{\Gamma \left(\left|\overset{⇀}{\alpha }\right|\right)}{\prod_{s=1}^{S}\Gamma \left(\alpha_{s}\right)}\frac{\prod_{s=1}^{S}\Gamma \left(n_{kjs}+\alpha_{s}\right)}{\Gamma \left(\left|{\overset{⇀}{n}}_{kj}|+|\overset{⇀}{\alpha }\right|\right)}\right) \end{array}$$

where $\overset{⇀}{a}=\left(a_{1},\ldots ,a_{S}\right)$ is set as the genome-wide proportion of each epigenetic state in all epigenomes and multiplied by 5, and |·| denotes the sum of all elements in a vector.

Similarly, we assume that each epigenome follows a multinomial distribution to be assigned to the *K* groups in Ω_*c*_. Let *m*_*kc*_ denote the number of epigenomes assigned to the *k*th group in Ω_*c*_. Using the Dirichlet($\overset{⇀}{1}$) prior, we express Pr(Ω_*c*_) as

(3) $$\begin{array}{l} \mathrm{Pr}\left(\Omega_{c}\right)=\Gamma \left(K\right)\frac{\prod_{k=1}^{K}\Gamma \left(m_{kc}+1\right)}{\Gamma \left(|{\overset{⇀}{m}}_{kc}|+K\right)} \end{array}$$

Given the CSP index variable {*M*_*j*_}, we do not have to infer the parameters *p*_*M*_*j*__ in (1). Instead, denoting by *o*_1_, …, *o*_*C*_ the count of each CSP in the genome, we again assume a Dirichlet($\overset{⇀}{1}$) prior to *p*_*M*_*j*__ and marginalize it out to obtain the final form of our model:

(4) $$\begin{array}{l} P\left(\Omega ,M|X\right)\propto \left(\prod_{j}\prod_{k=1}^{K}\frac{\Gamma \left(\left|\overset{⇀}{\alpha }\right|\right)}{\prod_{s=1}^{S}\Gamma \left(\alpha_{s}\right)}\frac{\prod_{s=1}^{S}\Gamma \left(n_{kjs}+\alpha_{s}\right)}{\Gamma \left(\left|{\overset{⇀}{n}}_{kj}|+|\overset{⇀}{\alpha }\right|\right)}\right)\\ \frac{\Gamma \left(C\right)}{\Gamma \left(|\overset{⇀}{o}|+C\right)}\overset{C}{\underset{c=1}{\prod }}\left(\Gamma \left(o_{c}+C\right)\Gamma \left(K\right)\frac{\prod_{k=1}^{K}\Gamma \left(m_{kc}+1\right)}{\Gamma \left(|{\overset{⇀}{m}}_{kc}|+K\right)}\right) \end{array}$$

### Model fitting

Starting from random initialization, we iteratively updated the CSP index {*M*_*j*_} and the epigenome group assignment, denoted by $\left\{I_{k}^{c}\right\}$, in each CSP. Given {*M*_*j*_} and $\left\{I_{k}^{c}\right\}$, all other variables in our model were deterministic. We updated {*M*_*j*_} at each position and $\left\{I_{k}^{c}\right\}$ for each epigenome in the *c*th CSP by conditioning on the current values of all other variables. Since {*M*_*j*_} and $\left\{I_{k}^{c}\right\}$ were integer-valued, we enumerated all possible values and calculated the corresponding likelihoods from the model (4). We then updated the model by maximization. We used simulated annealing in the first 50 iterations with an initial temperature set at 5 to alleviate local mode problems. We set the total number of CSPs *C* = 50 and the number of groups *K* = 5 per CSP. Although these hyper-parameters were fixed, some CSPs and their epigenome groups did not have instances in the data, since our Bayesian model penalized larger models when smaller models were sufficient to explain the data. To reduce computational cost, we used 5% randomly selected genome to train the model (4), which yielded 48 distinct CSPs. Except for the constitutive CSP, where all epigenomes were assigned into one group, the other 47 CSPs assigned 127 epigenomes into two or three groups.

### Patterns enriched in GWAS variants

The risk variants of a trait may fall into two disjoint categories: (1) variants enriched or depleted at loci carrying certain cell-type specificities and (2) variants independent of cell-type specificities. To study enrichment of CSPs at the risk variants, we can classify the variants into two groups. In group 1, the variants have an unknown probability π_*c*_ of co-occurring with CSPs Ω_*c*_ (*c* = 1, …, *C*). In group 2, the variants are nonspecific to the CSPs and thus have probability *p*_*c*_ of co-occurring with Ω_*c*_, where *p*_*c*_ is given by the model (4). We assume that each risk variant has probability *q* of being in group 1 and probability 1 − *q* of being in group 2. For each complex trait, let *A* = {*A*_1_, *A*_2_} denote the group index of all risk variants. Let {*M*_*j*_} denote the CSP index for the *j*th variant. Let *l*_1_ = *|A*_1_*|* and *l*_2_ = *|A*_2_*|* denote the numbers of risk variants in groups 1 and 2, and {*z*_1*c*_} and {*z*_2*c*_} denote the numbers of risk variants co-occurring with Ω_*c*_ in each group. We assign a Dirichlet(1,β) prior to *q* and a Dirichlet($\overset{⇀}{a}$) prior to {π_*c*_}, and we analytically integrate out *q* and {π_*c*_} to obtain.

(5) $$\begin{array}{l} P\left(\text{A},\text{M}|X,\Omega \right)\propto \frac{\Gamma \left(1+\beta \right)\Gamma \left(l_{1}+1\right)\Gamma \left(l_{2}+\beta \right)}{\Gamma \left(\beta \right)\Gamma \left(l_{1}+l_{2}+1+\beta \right)}\frac{\Gamma \left(C\right)}{\Gamma \left(|{\overset{⇀}{z}}_{1}|+C\right)}\prod_{c=1}^{C}\Gamma \left({\text{z}}_{1c}+1\right){p_{c}}^{z_{2c}}\left(\prod_{j\in A}\prod_{k=1}^{K}\frac{\Gamma \left(\left|\overset{⇀}{\alpha }\right|\right)}{\prod_{s=1}^{S}\Gamma \left(\alpha_{s}\right)}\frac{\prod_{s=1}^{S}\Gamma \left(n_{kjs}+\alpha_{s}\right)}{\Gamma \left(\left|{\overset{⇀}{n}}_{kj}|+|\overset{⇀}{\alpha }\right|\right)}\right) \end{array}$$

Inference of the model (5) is performed similarly to that of the model (4), where the variables that need to be updated were *A* and {*M*_*j*_}. The hyper-parameter β in the model (5) must be >1 to favor the null model of no enrichment. Empirically, we have found that β = 10 or the maximum number of disease variants in a linkage disequilibrium (LD) cluster (the set of variants that are in tight LD with a lead variant reported in GWAS; see the next section), whichever is greater, performs well in the sense that no enrichments are found under the null.

For each trait, we trained the model (5) on its risk variants and proxy variants. We obtained the list of variants assigned to group 1, which were enriched/depleted with respect to the CSPs and were used to calculate CSP enrichments. The variants assigned to group 2, on the other hand, were independent of the CSPs.

### Sets of disease risk variants

The disease-associated variants were obtained from the GWAS Catalog. We removed the traits with fewer than five lead variants. We used SNAP (Johnson et al., 2008) to identify proxy variants for the lead variants. We used the default setting of SNAP (1000 Genomes Pilot 1 SNPs in the CEU panel within 500 kb of the lead variant) and retained only the proxies with *r*^2^ > 0.95 with the lead variant (Supplementary Data). We included the proxy variants in our analysis for two reasons: (1) most lead variants reported in GWAS are likely noncausal, since they were selected based on maximum association signals that were confounded by allele frequencies and LD effects, and (2) including proxy variants increased the number of risk variants to be fitted in our predictive model.

### Calculating Z-scores for epigenetic state enrichment

At each variant, we have 127 epigenetic states in the 127 epigenomes. Let *n*_*s,g*_ denote the number of states *s* in the *g*th epigenome cluster as defined in Table 1, for *g* = 1, …, 10, and let *n*_*s*, −*g*_ denote the number of state *s* in the remaining nine clusters. Further, let *p*_*g*_ denote the proportion of epigenomes in group *g*. The *z*-score for state *s* in group *g* at a position is calculated as

$$\begin{array}{l} z=\frac{n_{sg}-(n_{sg}+n_{s,-g})p_{g}}{\sqrt{(n_{sg}+n_{s,-g})p_{g}\left(1-p_{g}\right)+1}} \end{array}$$

**Table 1:** Ten clusters of the 127 Roadmap Epigenomics epigenomes.

**Group**	**Epigenome mnemonic**
1	BLD.CD14.MONO, BLD.CD14.PC, BLD.CD15.PC, BLD.CD19.PPC, BLD.CD3.PPC, BLD.CD34.CC, BLD.CD4.CD25.CD127M.TREGPC, BLD.CD4.CD25I.CD127.TMEMPC, BLD.CD4.CD25M.CD45RA.NPC, BLD.CD4.CD25M.CD45RO.MPC, BLD.CD4.CD25M.IL17M.PL.TPC, BLD.CD4.CD25M.IL17P.PL.TPC, BLD.CD4.CD25M.TPC, BLD.CD4.MPC, BLD.CD4.NPC, BLD.CD56.PC, BLD.CD8.MPC, BLD.CD8.NPC, BLD.DND41.CNCR, BLD.MOB.CD34.PC.F, BLD.MOB.CD34.PC.M, BLD.PER.MONUC.PC, THYM.FET
2	ESC.4STAR, ESC.H1, ESC.HUES48, ESC.HUES6, ESC.HUES64, ESC.I3, ESDR.CD184.ENDO, ESDR.CD56.ECTO, ESDR.CD56.MESO, IPSC.15b, IPSC.18, IPSC.20B
3	BRN.FET.M, BRN.GRM.MTRX, ESC.H9, ESC.WA7, ESDR.H1.BMP4.MESO, ESDR.H1.NEUR.PROG, IPSC.DF.19.11, IPSC.DF.6.9
4	BLD.CD19.CPC, BLD.CD3.CPC, BLD.CD34.PC, BLD.GM12878, THYM
5	BLD.K562.CNCR, ESDR.H1.BMP4.TROP, ESDR.H1.MSC, GI.CLN.MUC, GI.CLN.SIG, GI.ESO, GI.RECT.SM.MUS, GI.S.INT, GI.STMC.GAST, GI.STMC.MUC, HRT.ATR.R, HRT.FET, HRT.VENT.L, HRT.VNT.R, KID.FET, LNG, LNG.NHLF, MUS.PSOAS, OVRY, PANC, PANC.ISLT, PLCNT.AMN, SKIN.NHDFAD, SKIN.PEN.FRSK.MEL.01, SPLN, VAS.AOR
6	ADRL.GLND.FET, BRN.NHA, BRST.HMEC, BRST.HMEC.35, BRST.MYO, CRVX.HELAS3.CNCR, LNG.A549.ETOH002.CNCR, MUS.HSMM, MUS.HSMMT, SKIN.NHEK, SKIN.PEN.FRSK.KER.02, SKIN.PEN.FRSK.KER.03, SKIN.PEN.FRSK.MEL.03, VAS.HUVEC
7	BONE.OSTEO, FAT.ADIP.DR.MSC, FAT.MSC.DR.ADIP, LNG.IMR90, MUS.SAT, SKIN.PEN.FRSK.FIB.01, SKIN.PEN.FRSK.FIB.02, STRM.CHON.MRW.DR.MSC, STRM.MRW.MSC
8	BRN.ANG.GYR, BRN.ANT.CAUD, BRN.CING.GYR, BRN.DL.PRFRNTL.CRTX, BRN.HIPP.MID, BRN.INF.TMP, BRN.SUB.NIG
9	BRN.CRTX.DR.NRSPHR, BRN.FET.F, BRN.GANGEM.DR.NRSPHR, ESDR.H9.NEUR, ESDR.H9.NEUR.PROG, GI.CLN.SM.MUS, GI.DUO.MUC, GI.RECT.MUC.29, GI.RECT.MUC.31, GI.STMC.MUS, LIV.HEPG2.CNCR, MUS.SKLT.F, MUS.SKLT.M, PLCNT.FET
10	FAT.ADIP.NUC, GI.DUO.SM.MUS, GI.L.INT.FET, GI.S.INT.FET, GI.STMC.FET, LIV.ADLT, LNG.FET, MUS.LEG.FET, MUS.TRNK.FET

### Predicting GWAS variants

In addition to the enrichment analysis, we used CSPs to predict risk variants from GWAS, including both the lead and the proxy variants. As a control, we randomly selected 11,786 dbSNPs from the UCSC browser as the null variants, with minor allele frequencies and dbSNP function predictions matched to those of the risk variants. We used SNAP in the same setting as described above to identify strong proxies for the null variants, and the final set of null variants consisted of 69,087 SNPs (Supplementary Data).

For each complex trait, we used a generalized linear model (GLM) to predict the risk vs. null variants. We trained the GLMs by using only 50% of the risk variants for each trait and 50% of the null variants. We then calculated the prediction accuracy using the remaining 50% of data. Prediction accuracy was calculated as the area under the curve (AUC) of the precision-recall values for the model. We repeated this procedure 10 times independently and obtained an average AUC for each trait (traits with fewer than five risk variants in either training or testing data were removed from the analysis). We did not use the receiver operating curves (ROCs) to measure power, because the number of risk variants was too small relative to the number of null variants in most traits. For the same reason, we did not use the conventional 10-fold cross-validation method.

The predictors in our model were constructed as follows. First, given the epigenetic states {*X*_·*j*_} at the *j*th variant, we calculated the log likelihood from the formula (2) with Ω_*c*_ for *c* = 1, …, *C*. This yielded *C* (=48) scores. Second, we calculated the log *z*-scores for state enrichment in the 10 epigenome clusters, which were transformed to sign(*z*)^*^ log(|*z*|+1). This yielded 25 × 10 = 250 enrichment scores. The enrichment scores were highly correlated. Therefore, we performed principal component analysis (PCA) on the 250 enrichment scores and retained the first 48 principal components (PCs). As a result, at each variant, we had 48 scores for cell-type specificity and 48 for state enrichment, which were used as predictors in our model. The PCA was performed using training data only, and the same PCs were used in the testing data to convert enrichment scores.

We further used the epigenetic states in all cell types as predictors in our GLM. Given 25 states per cell type, we had 25 × 127 = 3,175 predictors, using all of which would inevitably over-fit the data, and they were highly correlated. We again used PCA to identify the first 96 PCs as the predictors to be used in the GLMs. We chose 96 PCs so to match with the number of predictors in our first model.

As a third model, we used the states in each cell type separately as the categorical predictors to predict GWAS variants. We identified the cell type yielding the best prediction as the single best cell type for predicting GWAS variants.

### Power analysis

Using 2,473,120 SNPs from Morris et al. (2012), we randomly selected 100 causal SNPs according to the precision-recall curve for each complex trait, i.e., *x*% of causal variants were selected from the top *y*% SNPs as ranked by our predictive model, where *x* denotes 100^*^ recall and *y* was given by (100^*^ *x*/precision)/2,473,120. We first simulated the test statistics for all variants from *N*(0, 1) under a null model of no association. We then simulated the effect sizes λ for each causal variant from a normal distribution *N*(0.1, 0.05), which were then multiplied by −1 or +1 with 50% probability each to reflect protective or deleterious effects. The test statistic corresponding to effect size λ was calculated as $t=\left[log\left(1+\lambda \right)\right]/\sqrt{n}$ under the assumption that each variant can only explain a tiny proportion of the total disease variance, where *n* (=2,000) denotes sample size (1,000 cases and 1,000 controls). Due to LD among variants, we further added indirect association to all variants within 500 kb to the causal variant by $rt+\sqrt{1-r^{2}z}$, where *r* denotes the correlation between each variant and the causal variant, and *z* denotes the test statistic of the target variant under null model. This procedure yielded correlated test statistics among variants due to LD.

Let {π_*i*_} be the functional data-predicted probabilities for each variant being causal, and let *p*_cut_ denote a multiple testing adjusted threshold for significance (e.g., *p*_cut_ = 0.05). The marginal *p*-value threshold for each variant is then given by *p*_*i*_ = *p*_cut_π_*i*_/|π|. In this way, variants with high probabilities of being causal will receive liberal thresholds, and variants with low probabilities of being causal will receive stringent thresholds. We have previously shown (Zhang and Liu, 2011) that this approach can appropriately control the overall false-positive rate in the genome. Finally, power was calculated as the percentage of causal variants located within 1 kb of at least one detected significant variant.

## Results

### Identification of cell-type-specificity patterns

We used IDEAS (Zhang et al., 2016) to jointly infer a 25-state model in the 127 epigenomes from the Roadmap Epigenomics and ENCODE projects. The 25 states captured unique combinations of mean signals of histone marks, which corresponded to the signatures of distinct functional elements as previously verified by experiments. For instance, states with moderate H3K4me3 and high H3K4me1 indicate likely enhancer activities; states with high H3K4me3 and moderate H3K4me1 indicate likely promoter activities; states with high H4K36me3 indicate transcription activities; states with H3K27me3 indicate repressive activities; states with H3K9me3 indicates heterochromatin; and states with low signals in all histone marks indicate no activity. Using the 25-state model predicted across the genome and 127 epigenomes, we can study patterns of cell-type specificity with respect to their putative regulatory functions and use the functions to interpret disease variants.

**Figure 1:**
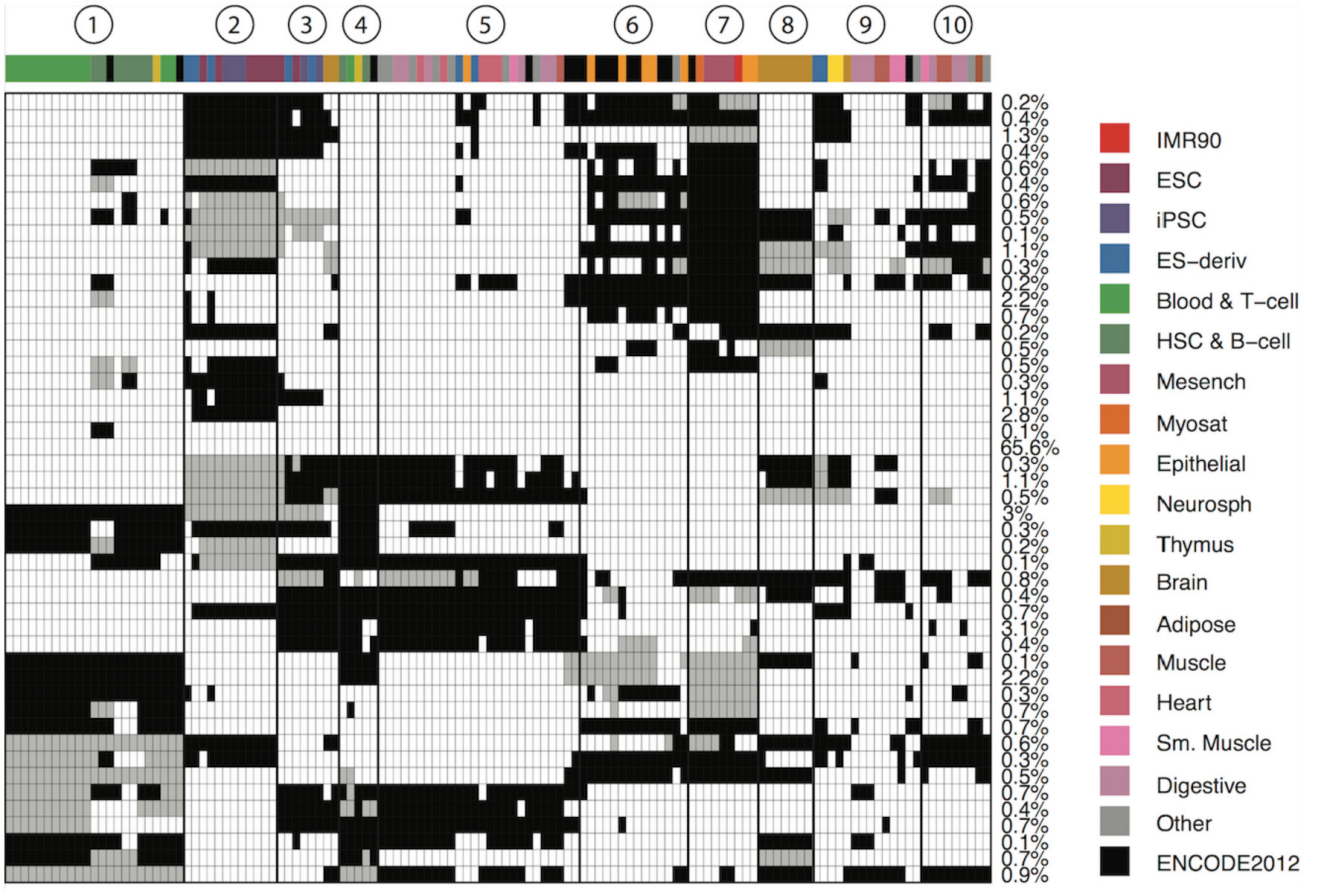
Genome-wide patterns of cell type specificity. X-axis denotes the 127 epigenomes, with epigenome color keys shown on the top and the right hand side. The cell type abbreviations are given by the Roadmap Epigenomics consortium. Y-axis shows the 48 CSPs. Each row in the matrix denote one epigenome partition pattern, with different groups of epigenomes indicated by black, white and gray. The percentage of the genome carrying each pattern is shown on the right. We also marked 10 clusters of epigenomes that were often together in most patterns.

We define a CSP as a partition of the 127 epigenomes such that epigenetic states in epigenomes within the same partition follow the same distribution, while states in epigenomes in different partitions follow different distributions. We developed a Bayesian algorithm to identify 48 major reoccurring CSPs in the genome (Supplementary Data), where each CSP represented a unique cell type-specificity pattern (Figure 1). One of the 48 CSPs had all epigenomes assigned to one group, which corresponded to non–cell-type-specific regions and occurred in 65.6% of the genome. This CSP constituted mainly low-signal regions or conserved transcription start sites. The remaining 47 CSPs had much lower abundances in the genome, with each occurring in about 0.1–3% of the genome. These 47 CSPs captured cell-type-specific regulatory events and thus are most interesting.

The 47 CSPs revealed roughly 10 distinct groups of epigenomes (Figure 1), which agreed well with the known cell-type origins (Table 1). For instance, most of the lymphocytes (Blood & T-cells and HSC & B-cells) were grouped together (cluster 1) in all CSPs, suggesting that the functional elements in these cell types are positively correlated across the genome. The pluripotent stem cells (ESCs, iPSCs, and ES-deriv) were commonly distributed in two groups (clusters 2 and 3), and the functional elements between the two groups were frequently different. We further obtained a group of ENCODE cell types and epithelial cell types (cluster 6), a group of mesenchymal stem cells and some of their differentiated cell types (cluster 7), a group of brain tissues (cluster 8), and a group of fetal tissues (cluster 10). Of the remaining epigenome groups, cluster 4 contained a small set of primary lymphocytes. Clusters 5 and 9 contained mixed cell types of different origins. Since we did not use the known cell-type origins as input, their agreement with our results confirmed that the inferred CSPs were reasonably accurate.

### Cell-type-specific enrichment in GWAS variants

**Figure 2:**
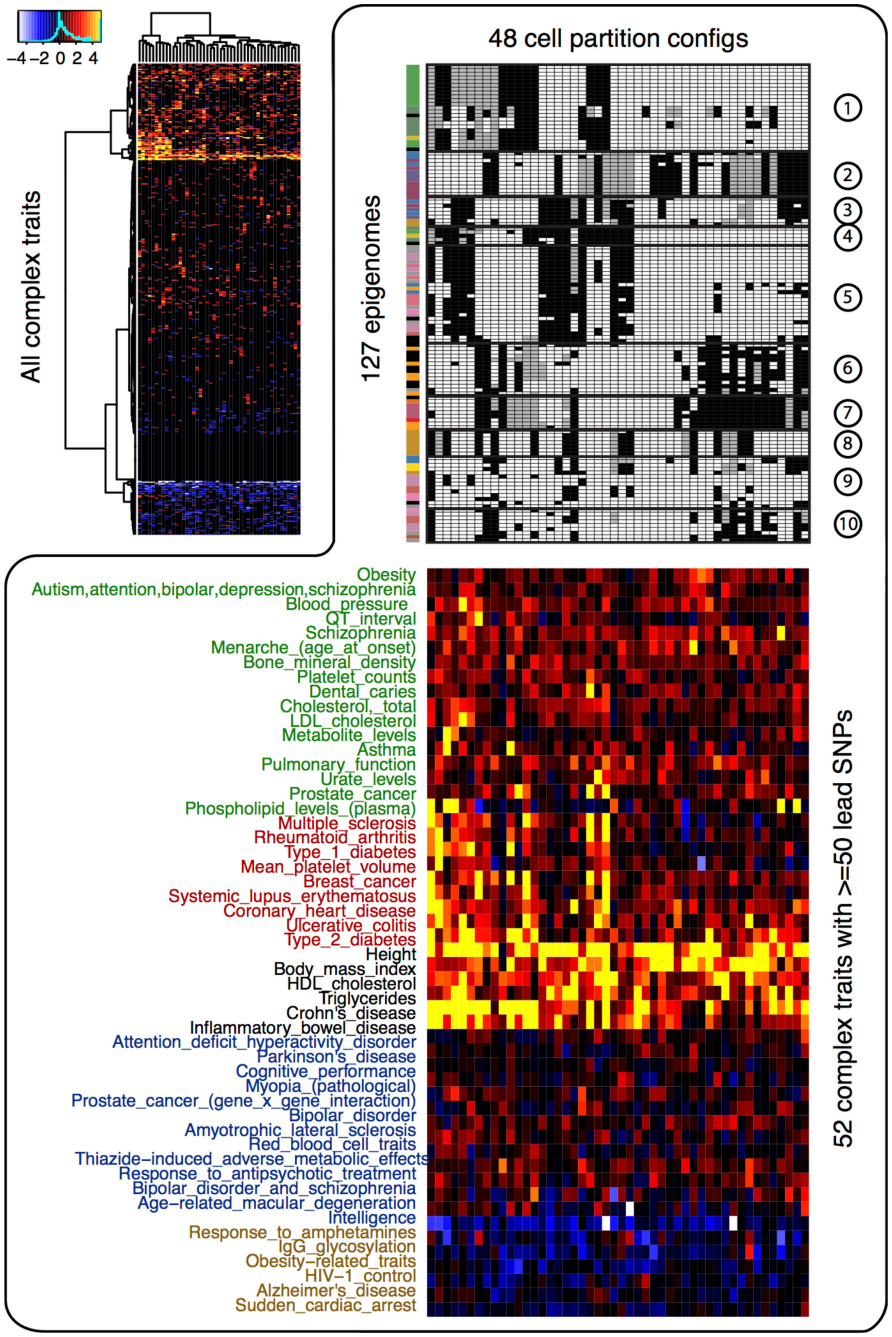
Enrichment of the 48 cell type specificity patterns in disease variants. The heatmap at top left corner shows the –log10 *p*-value of enrichment/depletion for the risk variants of all complex trait (y-axis) in each CSP (x-axis). Significant depletion is shown in blue and white (−log10 *p*-value is multiplied by −1 for depletion), and significant enrichment is shown in red and yellow. The heatmap at lower right corner shows the −log_10_ *p*-value of enrichment/depletion for the risk variants of 52 traits that have at least 50 lead variants. The upper right panel shows the 48 CSPs.

We next investigated the enrichment of the 48 CSPs in the disease variants. If the risk variants of a complex trait are enriched in some CSPs, then the corresponding epigenome partitions will inform us of potential functional relationships between the cell types and the trait. For all complex traits in the GWAS Catalog (Welter et al., 2014), we treated the lead GWAS variants and their strong proxy variants (LD *r*^2^ > 0.95) as the risk variants. We calculated two-sided permutation *p*-values (10,000 permutations) for the enrichment/depletion of those risk variants in each of the 48 CSPs. As shown in Figure 2, the enrichment/depletion of disease variants can be roughly categorized as (1) strongly enriched in all CSPs, (2) moderately enriched in a subset of CSPs, (3) enriched in specific CSPs, or (4) enriched in one or two CSPs and depleted elsewhere. For instance, physical traits (height, BMI, HDL, etc.) tended to be enriched in most CSPs, indicating that they may not be associated with specific cell types. Autoimmune diseases (multiple sclerosis, rheumatoid arthritis, type 1 diabetes, etc.), on the other hand, tended to be enriched in CSPs that uniquely clustered blood and immune cell types. A few mental and nerve-related disorders (Parkinson's disease, cognitive performance, bipolar disorder, intelligence, etc.) showed enrichment in a specific set of CSPs that highlighted brain-tissue specificity. In addition, Alzheimer's disease showed enrichment in monocytes.

We further evaluated whether specific functional elements are enriched in disease variants. Since most reported GWAS variants are likely tagging the true but unobserved causal mutations, we first used a Bayesian model to sub-select disease variants within each group of variants in strong LD, and we identified those variants showing significant enrichment in CSPs. About three-quarters of the traits in the GWAS Catalog had at least one risk variant enriched in CSPs (Supplementary Data). The proportion of enriched variants in each trait ranged from 0 to 76.3%, with a mean of 14.8%. For traits with CSP-enriched risk variants, their risks are likely affected by mutations in a cell-type-specific manner. On the other hand, for traits without CSP-enriched risk variants, their risk variants may not affect cell-type-specific regulation. The number of risk variants available from the GWAS Catalog did not bias our calculation, since the proportion of enriched risk variants in each trait was not associated with the number of risk variants for the trait (Pearson correlation 0.013).

**Figure 3:**
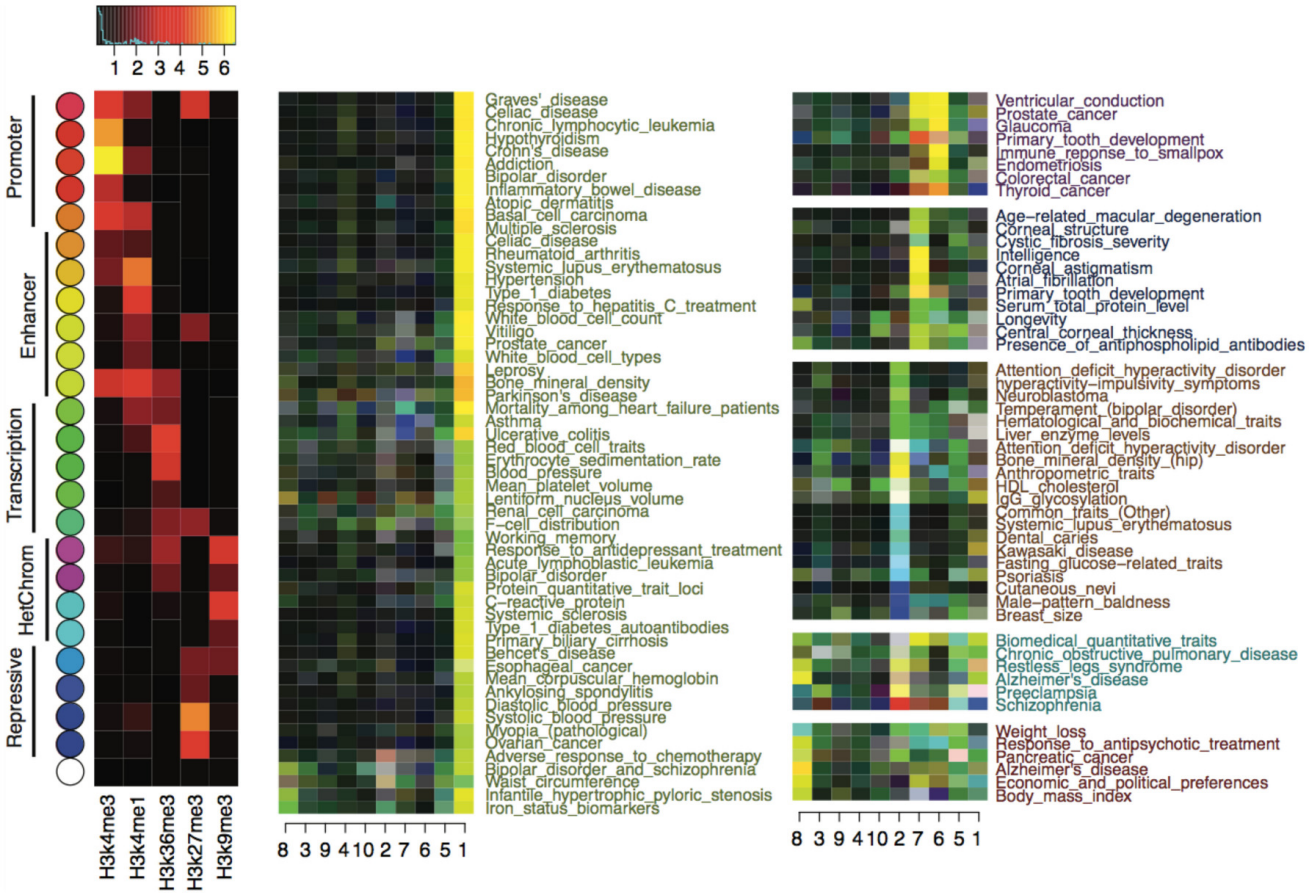
Enrichment of epigenetic states in disease variants with respect to the 10 epigenome clusters defined in Table 1. The left most heatmap shows the mean signal of histone marks in the 25 epigenetic states. State color keys and their putative functions are shown on the left. The right two panels show the enrichment of epigenetic states at the risk variants of each complex trait, with colors reflecting the most enriched states (given by the state color keys) and the strength of enrichments (brighter color means stronger enrichment).

We calculated *z*-scores for each trait using CSP-enriched risk variants to quantitatively measure the enrichment of epigenetic states at the risk variants with respect to the 10 epigenome clusters defined in Table 1. The *z*-scores were calculated at each risk variant and then averaged for each trait. We subtracted background *z*-scores using genome-wide null variants, and the final *z*-score matrix revealed interesting enrichment of epigenetic states. In particular, the epigenetic states labeling enhancers, transcriptions, and repressions were substantially enriched. As shown in Figure 3, for instance, autoimmune and blood-related traits exhibited enhancer (yellow) and transcription (green) enrichment exclusively in the blood cell types (cluster 1). Physical traits, including male baldness, breast size, psoriasis, dental caries, and common traits, had enriched enhancer, transcription, or repression (blue) activities in pluripotent stem cells (cluster 2). Another set of physical traits, including central corneal thickness, longevity, primary tooth development, and intelligence, showed enrichment of enhancer and transcription activity in mesenchymal stem cells (cluster 7). Interestingly, in brain tissues (cluster 8), we observed enriched enhancer activities for Alzheimer's disease, response to antipsychotic treatment, schizophrenia, and weight loss. There was barely any enrichment of promoter activities (red) in any epigenome clusters, which was consistent with our observation that promoter activities are highly conserved across cell types.

### Prediction of GWAS variants using cell-type-specificity patterns

Finally, we evaluated how well the epigenetic states and their CSPs can predict GWAS variants. We used the risk variants (without sub-selection) of each complex trait and a set of randomly selected null variants with matched minor allele frequencies and functional annotations as the response variable (binary). We used the log likelihood of the CSPs and the epigenetic state enrichment in the 10 epigenome groups in Table 1 as the predictor variables. For comparison, we also ran two other models to predict GWAS variants. One used the epigenetic states in all epigenomes as predictors, and the other used the epigenetic states in the best-matched single cell type, where the best-matched cell type was identified as the cell type yielding the best prediction. We used the AUC of the precision-recall plot to measure the prediction accuracy of each model for each complex trait.

**Figure 4:**
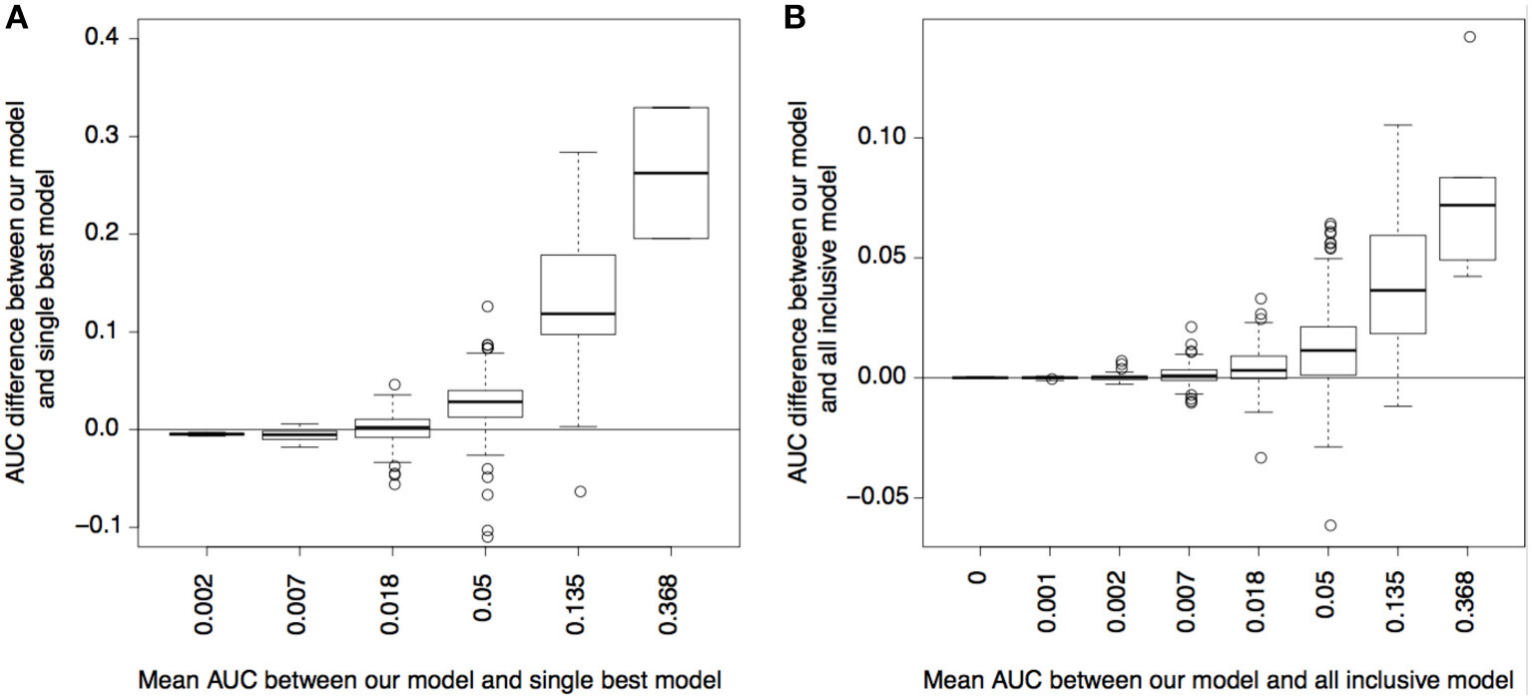
Power for predicting disease variants. **(A)** AUC difference of precision-recall curves between our model and single best cell type model (y-axis) plotted with respect to the mean AUC between the two models (x-axis); the mean AUC reflects how well the variants of a complex trait can be predicted overall. **(B)** is similar to **(A)**, but compares between our model and the model using a linear combination of all cell types.

As shown in Figure 4, using CSP and state enrichment scores produced substantially better predictions than using the best-matched single cell types, particularly for traits whose risk variants were overall predictable (as reflected by the mean AUC between methods). As expected, the power gain was partially due to the fact that we used more data than single cell types. However, for many traits, our method also outperformed the linear model using all cell types as input. Our result thus confirmed that the combinatorial relationships between functional elements captured by the CSPs could also increase prediction accuracy.

**Figure 5:**
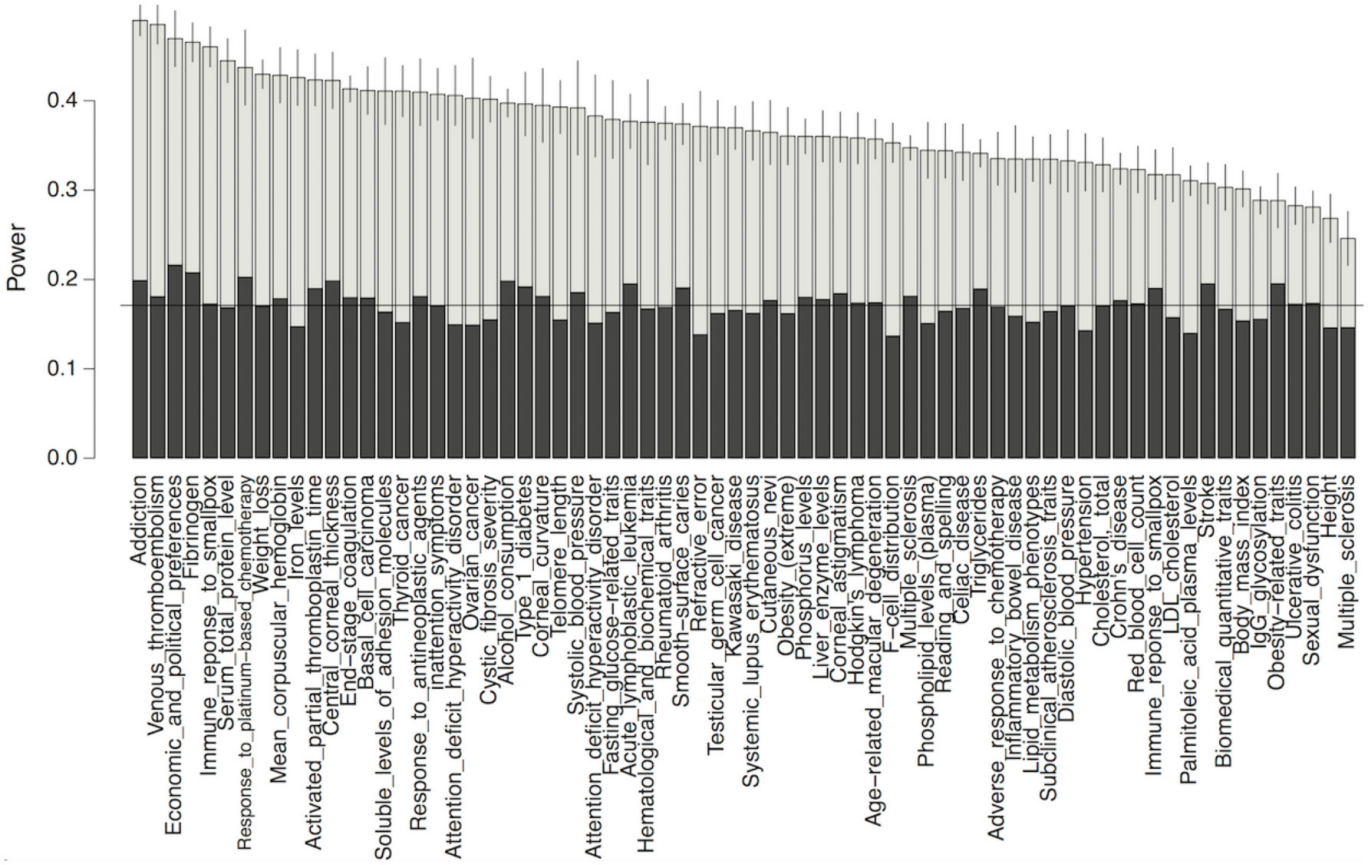
Power comparison between the conditional test method using functional priors (gray) and the fixed threshold method without priors (black) for detecting causal variants in GWAS. Horizontal line marks the mean power without using priors. Only the top 70 traits whose mean AUCs >0.05 in our model were shown.

To demonstrate the potential gain in power from using our predictions, we performed a power analysis for detecting association in GWAS on each complex trait, while using our functional data-predicted probability of being causal variants as a prior. As shown in Figure 5, our test using the functional priors uniformly boosted the power for detecting causal variants. Without using the priors, the mean power for detecting a causal variant in each trait was 17.2% for our simulation setting. After using the priors, the powers were increased to as much as 51%. As expected, the power gain was positively correlated with the prediction accuracy of our models (*p*-value 0.00156). It was also trait-dependent, since the traits had different precision recall curves even if their mean AUC-values were similar.

**Figure 6:**
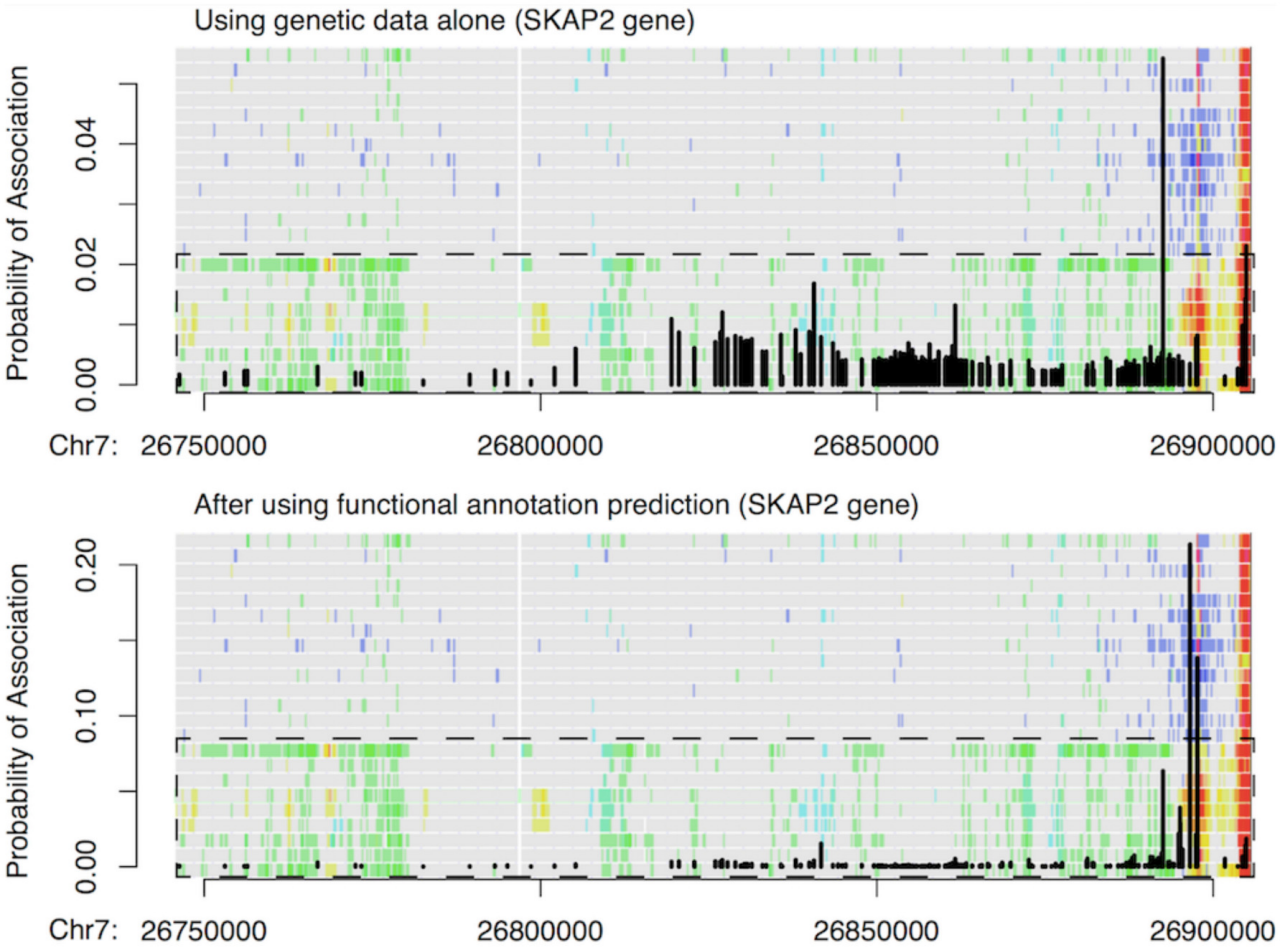
Example of change in IBD association probability before (top) and after (bottom) incorporating functional predictions at SKAP2 gene. Black vertical lines show the probability of IBD association at 179 credible variants obtained by Huang et al. (2015) via fine mapping using genetic data alone. The probabilities are overlaid with IDEAS functional annotation map to highlight how probabilities changed with functions. Overall, green in the functional annotation map indicates transcription, blue indicates repression, red indicates promoter activity, yellow/orange indicates enhancer activity, and gray indicates no regulatory events. The dashed box shows the group (rows) of blood T cells, and the remaining rows in the functional map are blood B & HSC cells.

Finally, we used 179 credible risk variants for inflammatory bowel disease (IBD) at the *SKAP2* gene from Huang et al. (2015) to demonstrate how our functional predictions can prioritize some of the low-probability IBD associations. As shown in Figure 6, a single variant within a transcribed region of *SKAP2* had the largest probability of association indicated by genetic data alone, while the remaining risk variants had flat but nontrivial probabilities of IBD association as well. This latter observation was due to strong LD within the region. After incorporating our functional predictions, two different variants stood out as being more likely to have an impact on IBD risk, since they were located within T-cell-specific enhancers. In contrast, the originally most significant variant and all other variants had much reduced probabilities of IBD association, including the variants located within the promoter region of *SKAP2*. This example thus highlights how our approach can borrow information from epigenetic CSPs to pinpoint potential functional variants that are otherwise undistinguishable when using genetic data alone.

## Discussion

We have introduced a computational approach to identify recurring patterns of cell-type specificity in the genome of 127 human cell types. By focusing on the co-occurrence patterns of epigenetic states, we have been able to use a small number of CSPs to explain most epigenomic variation in the genome. The corresponding epigenome partitions within CSPs agree well with known cell-type origins and are strongly enriched in the risk variants of many complex traits. The enrichment of active and repressive elements suggests both known and novel relationships between cell types and complex traits, and thus offers new insights for interpreting the regulatory effect of DNA mutations on disease risk in a cell-type-specific context. We have further demonstrated that using cell-type specificity could improve prediction of disease variants compared with using a linear model of functional elements alone.

The study presented here is complementary to existing work on utilizing functional data in fine mapping. Specifically, our approach is a computationally tractable method for detecting combinatorial patterns of functions across cell types, which can be included as additional predictors in existing methods to improve their power to prioritize disease variants. It should also be possible to use the functional data to perform conditional testing of disease association (Zhang and Liu, 2011), where a variant with weaker genetic association could be prioritized over other variants (with stronger genetic association) by using a more liberal threshold, if its functional information was more relevant to the disease.

There are a few limitations of the current study. First, we have exclusively focused on predicting disease variants from regulatory marks, which may lead us to miss disease mutations that directly affect protein coding. Our analysis included the H3K36me3 mark in the annotation, and thus the coding variants may be partially predictable by transcription states. It is, however, desirable to include additional and complementary genome annotation to improve prediction. Second, we used log *p*-values provided by the Roadmap Epigenomics project as input to our segmentation algorithm, by which data bias in different cell types should have been adjusted. The fact that we observed some epigenomic similarity between cell types from different origins, however, warrants more careful investigation. Third, a previous study suggested that 95% of the lead variants reported in the GWAS Catalog might not be causal (Farh et al., 2015). This limits our ability to detect epigenomic enrichment in the disease variants. We have alleviated this issue by including proxy variants as well as the lead variants, and we have developed a Bayesian method to sub-select candidate causal variants by explicitly assuming that not all reported variants are causal. However, because only a limited number of disease variants are available in the GWAS Catalog, we have only used logistic linear regression to predict the most likely causal variants. It would be desirable to improve the power further using nonlinear models and machine learning methods if a greater number of variants become available, for example by using whole-genome summary statistics from GWAS or combining the disease variants of closely related traits together via mixed effect models.

### URLs

The list of variants and the software tools used to generate the results in this paper are available in Supplementary Data. The IDEAS tool is available through the author's website at http://stat.psu.edu/~yuzhang/IDEAS/. Summary information on the 127 cell types is available from the Roadmap Epigenomics Consortium at https://docs.google.com/spreadsheet/ccc?key=0Am6FxqAtrFDwdHU1UC13ZUxKYy1XVEJPUzV6MEtQOXc&usp=sharing#gid=15.

## Author contributions

YZ conceived, designed, and implemented the study and wrote the manuscript.

### Conflict of interest statement

The author declares that the research was conducted in the absence of any commercial or financial relationships that could be construed as a potential conflict of interest.
